# Impacts of Precipitation and Temperature on Changes in the Terrestrial Ecosystem Pattern in the Yangtze River Economic Belt, China

**DOI:** 10.3390/ijerph16234872

**Published:** 2019-12-03

**Authors:** Jingwei Xiang, Weina Zhang, Xiaoqing Song, Jiangfeng Li

**Affiliations:** 1 School of Public Administration, China University of Geosciences, Wuhan 430074, China; xiangjw@cug.edu.cn (J.X.); jfli@cug.edu.cn (J.L.); 2 Hunan Key Laboratory of Land Resources Evaluation and Utilization, Changsha 410118, China; 3 School of Geography and Information Engineering, China University of Geosciences, Wuhan 430074, China; songxq@cug.edu.cn

**Keywords:** precipitation, temperature, terrestrial ecosystem pattern, impact utility, the Yangtze River Economic Belt

## Abstract

The terrestrial ecosystem plays an important role in maintaining an ecological balance, protecting the ecological environment, and promoting the sustainable development of human beings. The impacts of precipitation, temperature, and other natural factors on terrestrial ecosystem pattern change (TEPC) are the basis for promoting the healthy development of the terrestrial ecosystem. This paper took the Yangtze River Economic Belt (YREB) as the study area, analyzed the temporal and spatial characteristics of TEPC from 1995 to 2015, and used spatial transfer matrix and terrestrial ecosystem pattern dynamic degree models to analyze the area transformation between different terrestrial ecosystem types. A bivariate spatial autocorrelation model and a panel data regression model were used to study the impacts of precipitation and temperature on TEPC. The results show that: (1) The basic pattern of the terrestrial ecosystem developed in a relatively stable manner from 1995 to 2005 in the YREB, and transformations between the farmland ecosystem, forest ecosystem, and grassland ecosystem were more frequent. The temporal and spatial evolution of precipitation and temperature in the YREB showed significant regional differences. (2) There was a significant negative bivariate global spatial autocorrelation effect of precipitation and temperature on the area change of the forest ecosystem, and a positive effect on the area change of the settlement ecosystem. The local spatial correlation between precipitation or temperature and the terrestrial ecosystem showed significant scattered distribution characteristics. (3) The impacts of precipitation and temperature on TEPC showed significant regional characteristics on the provincial scale. The impact utility in the tail region is basically negative, while both positive and negative effects exist in the central and head regions of the YREB. Moreover, the impact showed significant spatial heterogeneity on the city scale. (4) The Chinese government has promulgated policies and measures for strategic planning, ecological environment protection, and economic support, which could effectively promote ecological and sustainable development of the YREB and promote the coordinated development of the ecology, economy, and society in China.

## 1. Introduction

The terrestrial ecosystem is the second largest carbon bank in the world behind the ocean, which is the key element of global climate change, and is of great significance for human development [1]. Terrestrial ecosystem pattern change (TEPC) and its impacts on global climate change and the ecological environment are some of the most concerning scientific issues at home and abroad in the 21st century, and have received much attention from governments, scientific communities, and the public. Precipitation and temperature are important natural factors affecting TEPC [2,3]. Changes in the temporal and spatial patterns of precipitation and temperature will affect the temporal and spatial distribution of water resources and nutrients, and then affect the terrestrial ecosystem carbon service, ecosystem function, ecosystem service value, economic development, human activities, and global climate change. Therefore, it is of great practical significance to reveal the impacts of precipitation and temperature on TEPC and to formulate strategies to deal with climate change under different ecological patterns, which is an important guarantee to promote regional ecological security protection and human development.

Scholars have studied TEPC and its influencing factors from different aspects, and a series of achievements have been obtained. Research on the terrestrial ecosystem mainly focuses on the value of ecological services [4], carbon fixation services [5], carbon reserves [6], carbon sequestration potential [7], mineral substances [8], biological elements [9,10], net primary productivity [11], and so on. The methods used mainly include process-based terrestrial ecosystem models such as IBIS (Integrated BIosphere Simulator) [10], remote sensing [12], mathematical methods [13], index systems [14], and machine learning approaches [15], etc. The research scale mainly involves the global scale [1], national scale [16], regional scale [17], and city scale [18]. 

As for the influencing factors of the terrestrial ecosystem pattern, they are mainly analyzed from the aspects of climate change [19], urban expansion [20], CO_2_ concentration [21], biodiversity [22], land use change [23] and land cover change [16], transfer of animals and plants [24], vegetation vulnerability [12], and so on. Based on these studies, several conclusions have been drawn. First, drought is the most important physical stress of terrestrial ecosystems [25]. Further, different terrestrial system compartments vary in stable mineral elements [8]. Temperature is the dominant factor influencing the respiratory process of terrestrial ecosystems [3]. Finally, the constraint effects between paired terrestrial ecosystem services and associated key features are shaped by climate factors, NDVI (normalized difference vegetation index), and other factors [26]. Thus, a solid foundation for research on the influencing factors of the terrestrial ecosystem is established. In terms of the study of the relationship between temperature, precipitation, and the development of the terrestrial ecosystem pattern, it is mainly focused on carbon exchanges and their responses to temperature and precipitation [17], net primary productivity allocation under different precipitation conditions across global grassland ecosystems [27], and precipitation altering temperature effects on ecosystem respiration in alpine meadows [28], etc.

The Yangtze River Economic Belt (YREB) is an important ecological security barrier in China, and also an important terrestrial ecosystem research area. At present, research on the terrestrial ecosystem of the YREB is mainly related to ecosystem services, carbon emissions, etc. Examples include eco-efficiency [29], the identification of ecological red lines [30], ecosystem service trade-offs and determinants [31], the real “green” way (i.e., a sustainable way or a way with minimal environmental impact) [32], policy impacts on local ecosystem services [33], efficiency, environmental regulation opportunity costs, and interregional industrial transfers [34], water footprint allocation [35], and carbon emission efficiency [36], etc. However, there are still some deficiencies in the impacts of precipitation and temperature on the terrestrial ecosystem of the YREB. At present, the development of the ecological environment of the Yangtze River is faced with many problems, such as the overall protection of the Yangtze River Basin being poor, the ecosystem being broken, and the service function being degraded. The environmental pollution is serious, and the ecological damage to the water environment is large. Contradiction between the development and protection of some areas is prominent, and regional development is not balanced. These problems seriously impede the sustainable and healthy development of the YREB. As we all know, precipitation and temperature are the important, foundational and natural influencing factors of the terrestrial ecosystem [2,3]. Research on the impacts of temperature and precipitation on the pattern change of the terrestrial ecosystem in the YREB brings not only an important contribution to clarify the influencing factors of the pattern change of the terrestrial ecosystem, but also puts forward ecosystem protection strategies for the YREB, and for the ecological environment improvement of the Yangtze River. Moreover, it is of great practical significance to promote the sustainable and healthy development of the social economy of the Yangtze River.

In China, the YREB plays an important strategic role in ecological protection and economic development. On the one hand, because of the large altitude differences, diverse topography and geomorphology, and complex climate types, a complex and diverse group of terrestrial ecosystem types is formed, such as farmland, forest, grassland, water, settlements and so on, which is an important ecological security barrier for China and even for the world, and can impact on human activities and national climate change. The concept of the YREB is restoring the “Yangtze River ecological environment” rather than “large-scale development” [31], and the Chinese government has formulated a series of policies to implement this concept, such as the “Outline for the Development Plan of the YREB (2016)” and the “Environmental Protection Plan for the YREB (2017)” [33], so as to promote the sustainable development of the ecological environment in the YREB. On the other hand, the YREB is not only the inland river economic belt with the most global influence in China, as well as a coordinated development zone of interaction and cooperation between the east and the west of China, but it is also the internal and external opening zone along the Yangtze River in an all-round way, giving it a remarkable economic strategic position, and now it has become one of the new ongoing national strategies with the One Belt and One Road initiative [33]. 

The ecological capacity of different ecosystem types is different, the value of ecological services is different, and the carbon storage is also different. Precipitation and temperature will affect TEPC, cause changes in ecological service value and carbon storage, and then affect the changes in the natural environment of the YREB, the sustainable development in China, and even sustainable development all over the world. Therefore, it is of great practical significance to study the change characteristics of the terrestrial ecosystem pattern and the impacts of precipitation and temperature on it in the YREB, which is of great practical significance to promote the balanced and healthy development of the terrestrial ecosystem in the YREB, and to promote the construction of ecological civilization in China, the rapid and sustainable development of the economy, harmony and stability of society, and the sustainable progress of human beings.

In view of this, this paper takes the YREB, an important development strategic area in China, as the research object, focusing on two problems: (1) the temporal and spatial differentiation characteristics of TEPC in the YREB for the years 1995, 2000, 2005, 2010, and 2015; (2) the effects of precipitation and temperature on TEPC in the YREB. So as to clarify TEPC and its influencing factors in the YREB, we aim to provide relevant suggestions for the protection of the ecological environment and the construction of ecological civilization in the YREB, and to provide scientific reference for promoting the sustainable utilization of natural resources and human development.

## 2. Materials and Methods

### 2.1. Study Area

**Figure 1:**
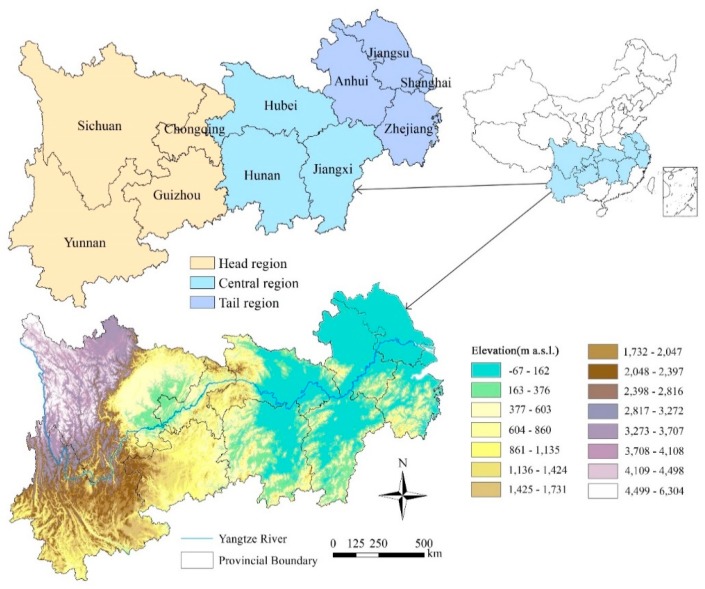
Location of the Yangtze River Economic Belt (YREB). The digital elevation model (DEM) was downloaded from the Resource and Environment Science Data Center of the Chinese Academy of Sciences [39]. The YREB can be divided into three parts: the head, central, and tail regions. The central region includes three provinces: Hubei, Hunan and Jiangxi.

The YREB (21°8′–35°20′N, 92°21′–123°10′E) can be divided into three regions: the head region (including Chongqing city, and Sichuan, Yunnan and Guizhou provinces), the central region (including Hubei, Hunan and Jiangxi provinces) and the tail region (including Shanghai city, and Jiangsu, Zhejiang and Anhui provinces), covering a total of 11 provinces and cities [37] (Figure 1). The YREB covers an area of about 2.05 million square kilometers, accounting for 21% of China, and the population and economy exceed 40% of China [31]. 

In terms of economic development, the YREB is a giant economic belt that connects different types of regions in the east and west of China. It is also the basin economic belt with the largest population, the largest industrial scale and the most complete urban system in the world. It plays a very important strategic role in national environmental protection, economic development and new urbanization. In terms of ecological environment, the YREB straddles the tropics, subtropical and warm temperate zones, with complex geomorphological types and diverse ecosystem types. The forest coverage rate of the Yangtze River Basin is 41.3%. The area of rivers, lakes, reservoirs and wetlands accounts for about 20% of China’s total area. It is rich in species and resources and is an important ecological treasure house. At the same time, it is extremely rich in water resources and is the strategic water source of China. The average total amount of water resources for many years is about 995.8 billion cubic meters, accounting for 35% of the total water resources of the whole country, which ensures the domestic and productive water demand of 400 million people along the Yangtze River. What is more, it has important functions for soil and water conservation and flood regulation and storage, and is an ecological security barrier area [38]. 

However, the ecological environment of the YREB is currently facing many problems: the overall protection of the basin is insufficient, the ecosystem is broken, the ecosystem service function is degraded, and the water ecological environment is becoming more and more threatened [38]. How to promote the YREB to achieve an ecological priority and green development way is an important problem to be solved urgently in China. A basic premise for solving this important problem is to clarify the effects of natural factors such as precipitation and temperature on the terrestrial ecosystem pattern of the YREB.

### 2.2. Research Framework

**Figure 2:**
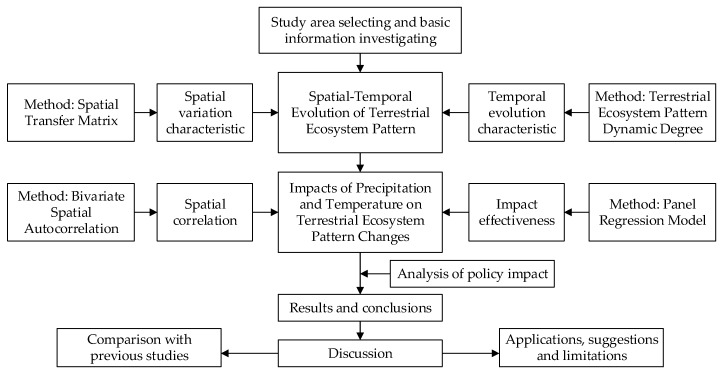
Research framework of this study.

Figure 2 depicts the framework of this study. Firstly, after selecting the study area, basic information was investigated for subsequent study. Secondly, the spatial variation characteristic was studied by using the spatial transfer matrix (STM) model, and the temporal evolution characteristic was studied by taking the terrestrial ecosystem pattern dynamic degree (TEDD) model, which is derived from the land use dynamic degree (LUDD) model. Thirdly, by using the bivariate spatial autocorrelation (BSA) method and the panel regression model (PRM), the impacts of precipitation and temperature on terrestrial ecosystem changes are analyzed in terms of spatial correlation and impact effectiveness. Lastly, after completing the policy impact analysis, conclusions are obtained mainly from the aspect of the spatial-temporal changes in the terrestrial ecosystem pattern and the impacts of precipitation and temperature on it. Discussions are elaborated from the aspects of comparison with previous studies, applications, suggestions, and limitations.

### 2.3. Methods

#### 2.3.1. Spatial Transfer Matrix

Drawing lessons from the related knowledge of the land use transfer matrix [40], this paper analyzes TEPC on the provincial scale, describes its structural characteristics, and reflects the transfer direction between different terrestrial ecosystem types guided by human activities. At the same time, a two-dimensional transition table is drawn to clearly express the area transition relationship between terrestrial ecosystem types in different periods of the YREB, so as to clarify the temporal and spatial evolution process of terrestrial ecosystem areas in a certain period.

The spatial transfer matrix can describe the structural characteristics of regional terrestrial ecosystem change comprehensively and concretely and reflect the direction of terrestrial ecosystem change guided by human activities. This method comes from the quantitative description of system state and its transition in system analysis. It reflects the transition process of a sub-stable system from the time *T* to *T*+1 at a certain time interval. Based on this, the temporal and spatial evolution process of the terrestrial ecosystem pattern can be better revealed [40]. Its mathematical expression is as follows:

(1) $$S_{ij}=\left[\begin{array}{cc} \begin{array}{cc} S_{11} & S_{12}\\ S_{21} & S_{22} \end{array} & \begin{array}{cc} \cdots & S_{1n}\\ \cdots & S_{2n} \end{array}\\ \begin{array}{cc} \cdots & \cdots \\ S_{n1} & S_{n2} \end{array} & \begin{array}{cc} \cdots & \cdots \\ \cdots & S_{nn} \end{array} \end{array}\right]$$

where *S_ij_* is the state of the terrestrial ecosystem at the beginning and end of the study period, and *n* is the number of terrestrial ecosystem types.

#### 2.3.2. Terrestrial Ecosystem Pattern Dynamic Degree

According to the related methods of land use dynamic degree (LUDD) [41,42], the dynamic degree of terrestrial ecosystem pattern (TEDD) is studied in order to quantitatively describe the change rate of terrestrial ecosystem areas in the YREB and clearly reflect its changing trend. This method includes a single type of TEDD and a comprehensive TEDD. The former represents a certain type of TEPC in a certain period, and the latter represents the comprehensive change of various types of terrestrial ecosystem area in a certain period. The expressions are as follows:

(2) $$K=\frac{U_{b}-U_{a}}{U_{a}}\times \frac{1}{T}\times 100\%$$

(3) $$LC=\left[\frac{\sum_{i=1}^{n}\Delta LU_{i-j}}{2\sum_{i=1}^{n}\Delta LU_{i}}\right]\times \frac{1}{T}\times 100\%$$

where *K* represents a certain type of TEDD, and U_a_ and U_b_ represent the areas of the certain type of terrestrial ecosystem at the beginning and end of the study period, respectively. *LC* is a comprehensive TEDD of various types. ΔLU*_i_* is the area of terrestrial ecological type *i* in the initial stage, ΔLU*_i-j_* is the absolute value of terrestrial ecosystem type *i* converted into other terrestrial ecosystem types during the study period, and *T* is the study period. 

#### 2.3.3. Bivariate Spatial Autocorrelation

The bivariate spatial autocorrelation (BSA) model [43] is used to study the spatial correlation between precipitation and temperature and the terrestrial ecosystem pattern in the YREB. This method is extended on the basis of the spatial autocorrelation model [44] and is divided into two parts: global spatial autocorrelation and local spatial autocorrelation. The former is used to describe the average correlation degree, spatial distribution pattern and significance of all objects in the whole research area. The latter is used to identify the possible spatial association patterns in different spatial positions, to clarify the local instability and heterogeneity of space, to grasp the aggregation and differentiation characteristics of local spatial elements more accurately, and to provide a basis for classification and decision making. The global spatial autocorrelation is calculated by Moran’s *I* index:

(4) $$I=\frac{n}{\sum_{i=1}^{n}\sum_{j=1}^{n}W_{ij}}\times \frac{\sum_{i=1}^{n}\sum_{j=1}^{n}W_{ij}\left(x_{i}-\bar{x}\right)\left(x_{j}-\bar{x}\right)}{\sum_{i=1}^{n}{\left(x_{i}-\bar{x}\right)}^{2}}$$

For a single spatial unit *I*, the local spatial autocorrelation can be studied, and the formula is as follows:

(5) $$I_{i}=\frac{n\left(x_{i}-\bar{x}\right)}{\sum_{i=1}^{n}{\left(x_{i}-\bar{x}\right)}^{2}}\cdot \overset{n}{\underset{j=1}{\sum }}W_{ij}\cdot \left(x_{j}-\bar{x}\right)$$

where *x_i_* and *x_j_* represent the attribute values of the spatial units *i* and *j*, respectively, *n* is the number of spatial units, and *W_ij_* is the weight matrix established based on the spatial adjacency relationship. The value of *I* is generally between −1 and 1. A value greater than 0 indicates a positive correlation, with relative agglomeration of spatial distribution, less than 0 indicates a negative correlation, where spatial distribution is relatively scattered, and equal to 0 means there is no spatial autocorrelation. *P* values are used for the significance test. In the calculation results, High-High (Low-Low) indicates that the spatial difference is small, and the attribute value is higher (lower) than the surrounding county and city. Low-High (High-Low) indicates that the spatial difference is large, the research unit attribute value is lower (higher), and the surrounding counties and cities are higher (lower).

Based on this, the formula of bivariate spatial autocorrelation is extended as follows:

(6) $$I_{lm}^{p}=\frac{X_{l}^{p}-\bar{X_{l}}}{\sigma_{l}}\cdot \overset{n}{\underset{q=1}{\sum }}(W_{pq}\cdot \frac{X_{m}^{q}-{\bar{X}}_{m}}{\sigma_{m}})$$

where $X_{l}^{p}$ is the attribute *l* value of spatial unit *p*, $X_{m}^{q}$ is the attribute *m* value of spatial unit *q*, $\bar{X_{l}}$ and ${\bar{X}}_{m}$ are the average values of attributes *l* and *m*, and $\sigma_{l}$ and $\sigma_{m}$ are the variance of attributes *l* and *m*, respectively.

#### 2.3.4. Panel Regression Model 

Taking 133 cities in the YREB as research units, the impact of precipitation and temperature on the terrestrial ecosystem pattern of the YREB is studied by using the panel regression model, which has the following advantages to influence measurement: (1) it does not need too large a sample capacity; (2) the development relationship between economic variables can be measured accurately; (3) it is possible to estimate the influence of certain difficult factors on the interpreted variables; (4) it can reduce the multiple collinearity between the explanatory variables and improve the estimation accuracy of the econometric model; (5) panel data usually combine section data and time variables, which reduces the problem caused by variable default. The specific calculation formula of the model is:

(7) $$ECO_{it}=\mu_{it}+\beta_{1,it}PRE_{1,it}+\beta_{2,it}TEM_{1,it}+\varepsilon_{it}$$

where ECO*_it_* indicates the area of the terrestrial ecosystem, PRE_l,*it*_ represents the value of precipitation, TEM_1,*it*_ represents the value of temperature, μ*_it_*, β_1,*it*_, β_2,*it*_ are coefficients to be estimated, *i* is the area number (*i* = 1, 2, 3,…, 133), *t* represents the year (*t* = 1995, 2000, 2005, 2010, 2015), and ε*_it_* represents a random interference term. In order to eliminate the influence of heteroscedasticity, the variables are logarithmic before they are calculated.

### 2.4. Data Source and Processing

The years of study in this paper were chosen as 1995, 2000, 2005, 2010, and 2015. The change in the land ecosystem pattern is a slow change process that is affected by many factors, such as climate, society, economy, population, etc. There is little change in the short term, the variation between adjacent years is small, and the characteristics of the terrestrial ecosystem are not significant. In addition, the five time points selected in this study can clearly show the change characteristics of temperature, precipitation and terrestrial ecosystem pattern at different times, which also meets the requirements of the mathematical models of panel regression and space self-correlation utilized in this paper. Therefore, the five data years of 1995, 2000, 2005, 2010, and 2015 were selected.

The raster data of terrestrial ecosystem types in the YREB are derived from the Resource and Environment Science Data Center of the Chinese Academy of Sciences [39], which is based on 1:100000 land use/land cover data obtained by remote sensing interpretation to identify, study and reclassify each ecosystem type, forming a 1 × 1 km dataset of Chinese terrestrial ecosystem types, including the farmland ecosystem, forest ecosystem, grassland ecosystem, water ecosystem, settlement ecosystem, and desert ecosystem among others. Because the proportions of the desert ecosystem and other ecosystem types are lower, they are not considered, and only the previous five types of terrestrial ecosystem are taken as the research object. It is worth noting that this paper is mainly based on the classification standards of China for the division of terrestrial ecosystems, which is different from that of other countries. For example, forest types in China include trees, shrubs, bamboo, coastal mangroves and so on, while Europe does not contain bamboo, and types are divided into herbs.

The annual precipitation and temperature data of the YREB also come from the Resource and Environment Science Data Center of the Chinese Academy of Sciences [39], and are based on the daily observation data of more than 2400 meteorological stations in China. A 1 × 1 km dataset is generated by sorting, calculating, and the spatial interpolation processing. In the process of calculation, the average value of all raster data in the area is taken as the precipitation or temperature value of the region, the province or city is considered as the study unit, and the mean value of all the raster data in the unit is taken as the precipitation or temperature value. 

## 3. Results

### 3.1. Spatial-Temporal Evolution of Precipitation and Temperature

#### 3.1.1. Spatial-Temporal Evolution of Precipitation

Table 1 shows the precipitation changes in three regions of the YREB for 1995, 2000, 2005, 2010 and 2015. According to the law of time development, the precipitation in the whole YREB and its central and tail regions showed a fluctuating growth trend, and the fluctuation range in the central region of the YREB is relatively large, while that of the head region of the YREB showed a decreasing trend of fluctuation. The average precipitation in the central region was the highest (1557 mm), followed by 1325 mm in the tail area, and in the head region it was the lowest (1168 mm). Combined with the digital elevation model (DEM) showed in Figure 1, it can be found that the altitude of different basins of the Yangtze River has a certain impact on the precipitation, and the altitude from the head region to the tail region decreases gradually from 6304 m to sea level; especially in western Sichuan, the precipitation is relatively small. Most of the central region is plains, such as Jianghan Plain and Dongting Lake Plain, which are famous granaries in China, and the rich precipitation ensures high grain output.

**Figure 3:**
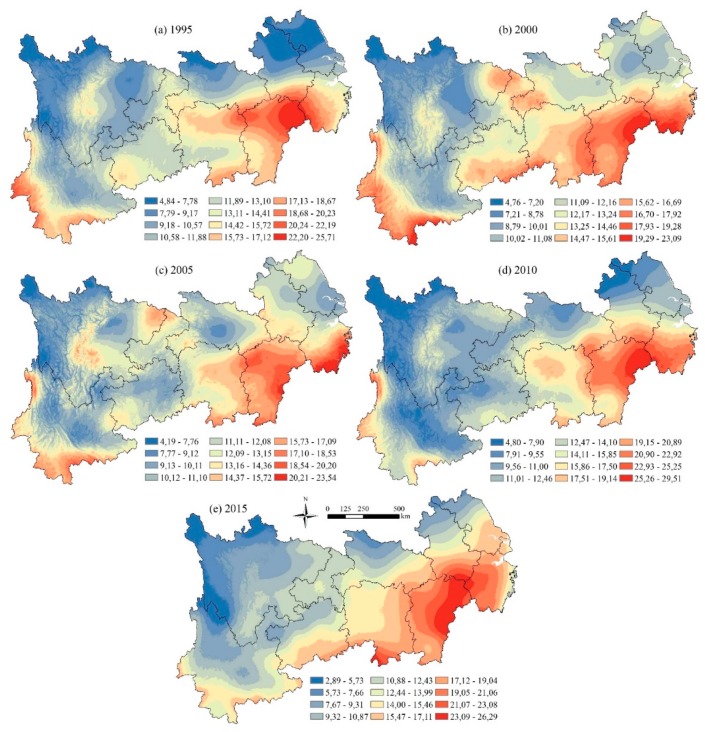
The spatial distribution of precipitation in the YREB (mm). (**a**) Spatial distribution of precipitation in 1995; (**b**) Spatial distribution of precipitation in 2000; (**c**) Spatial distribution of precipitation in 2005; (**d**) Spatial distribution of precipitation in 2010; (**e**) Spatial distribution of precipitation in 2015.

Figure 3 shows the spatial distribution of precipitation in the YREB for 1995, 2000, 2005, 2010 and 2015. The areas with high precipitation are mainly distributed in Jiangxi Province in the central and tail regions of the YREB, the eastern part of Hunan Province, the southwest of Zhejiang Province, and the southern part of Yunnan Province. The regions with low precipitation are in the head region of Sichuan, and central and northern Yunnan, and the northern areas of Anhui and Jiangsu also had low precipitation in 1995 and 2010. Generally speaking, the precipitation in the YREB shows spatial distribution characteristics of high in the southeast and low in the northwest.

#### 3.1.2. Spatial-Temporal Evolution of Temperature

Table 2 shows the temperature changes in the YREB and its head, central and tail regions for 1995, 2000, 2005, 2010, and 2015. The average temperatures in the central region are the highest, and are on the rise year by year, reaching 17.5 °C in 2015, followed by 16.4 °C in the tail region, while the average annual temperature in the head region is the lowest, at only 14.2 °C. On the whole, the average annual temperature of the YREB is 15.9 °C. From the point of view of fluctuation law, in addition to the obvious fluctuation characteristics in the tail region, the head and central regions show a certain upward trend of fluctuation. According to the analysis of the reasons, except for the different altitudes of the head, central and tail regions, the different economic structures and the intensity of human activities in each region are important reasons that affect the temperature difference.

**Figure 4:**
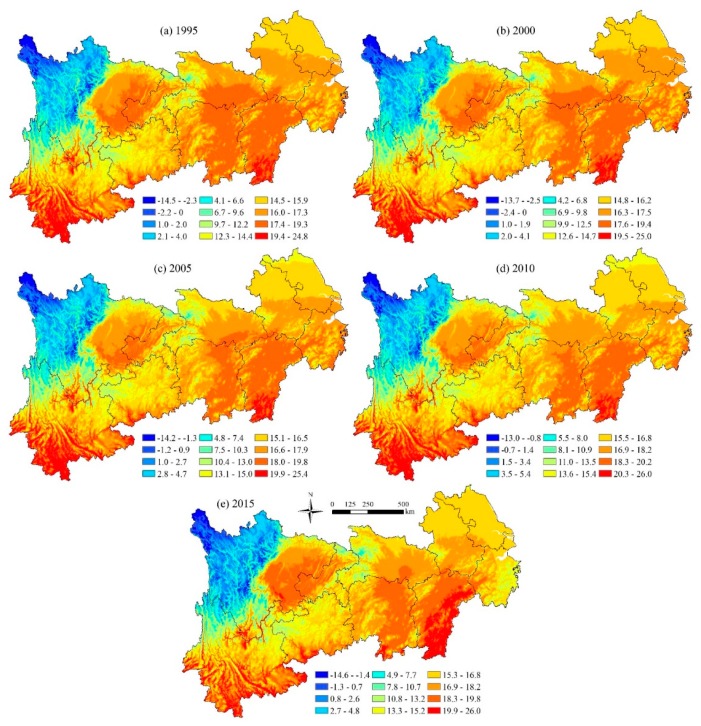
The spatial distribution of temperature in the YREB (°C). (**a**) Spatial distribution of temperature in 1995; (**b**) Spatial distribution of temperature in 2000; (**c**) Spatial distribution of temperature in 2005; (**d**) Spatial distribution of temperature in 2010; (**e**) Spatial distribution of temperature in 2015.

Figure 4 shows the spatial distribution of temperature in the YREB for 1995, 2000, 2005, 2010 and 2015. On the whole, the temperature of the YREB is low in the northwest and high in the southwest and southeast. The average temperature in western Sichuan and northern Yunnan is relatively low, and the areas with high temperature are mainly distributed in Yunnan Province and southern Jiangxi Province, southern Hubei Province, and eastern Hunan Province. Latitude and altitude are the important reasons that affect the spatial distribution of temperature. Generally speaking, the lower the latitude, the higher the altitude and the lower the temperature, which accords with the current characteristics of temperature spatial distribution in the YREB.

### 3.2. Spatial-Temporal Evolution of the Terrestrial Ecosystem Pattern

#### 3.2.1. Spatial Variation Characteristics

**Figure 5:**
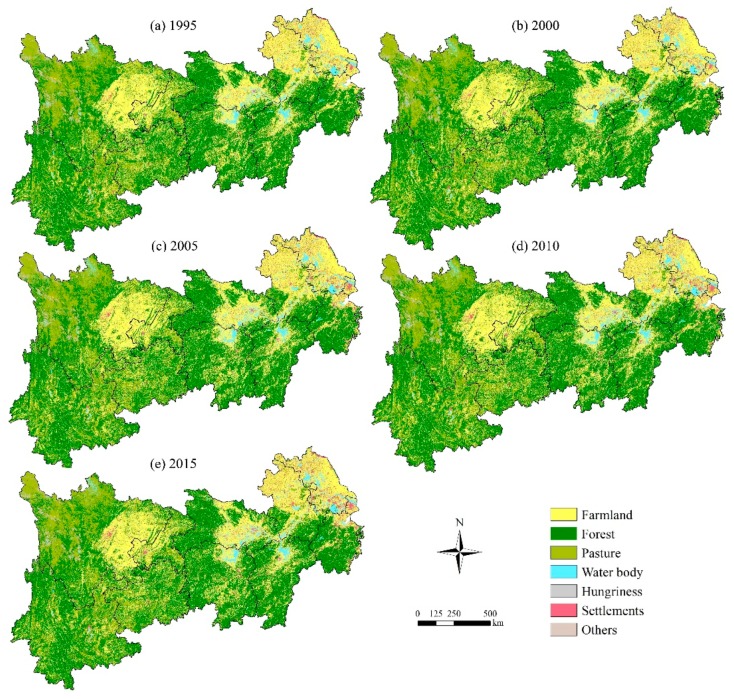
Spatial distribution of terrestrial ecosystem types in the YREB. The raster spatial data were downloaded from the Resource and Environment Science Data Center of the Chinese Academy of Sciences [39]. (**a**) Spatial distribution of terrestrial ecosystem types in 1995; (**b**) Spatial distribution of terrestrial ecosystem types in 2000; (**c**) Spatial distribution of terrestrial ecosystem types in 2005; (**d**) Spatial distribution of terrestrial ecosystem types in 2010; (**e**) Spatial distribution of terrestrial ecosystem types in 2015.

Figure 5 shows the spatial distribution of terrestrial ecosystem types in the YREB. On the whole, the basic distribution pattern of terrestrial ecosystem types in the YREB does not change much each year. Firstly, the area of the forest ecosystem is the largest, accounting for more than 50%, which is mainly distributed in the western and southern regions, and the province with the largest area is Yunnan Province. Secondly, the farmland ecosystem, accounting for about 30% of the total terrestrial ecosystem, is mainly distributed in the eastern and central regions of the YREB, including the Sichuan Basin, Jianghan Plain, and the middle and tail regions of the Yangtze River plain. The grassland ecosystem is mainly distributed in Sichuan Province, among which the northwest of Sichuan Province is the most widely distributed. Thirdly, the settlement ecosystem is obviously distributed in Shanghai, Jiangsu and other places in the east of the YREB, and the area tends to expand gradually. In addition to the Yangtze River, the water ecosystem is mainly distributed in Hubei, Hunan, Jiangxi, Jiangsu, and other places in the central and tail regions.

The spatial transfer matrix between terrestrial ecosystem types in the YREB for 1995, 2000, 2005, 2010 and 2015 is shown in Table 3. In different stages of development, the transfer paths and characteristics of terrestrial ecosystem types are not the same, showing a certain stage of development characteristics. Overall, during 1995–2015, transformations between the farmland ecosystem, forest ecosystem and grassland ecosystem are more frequent. The area of the farmland ecosystem increased by 20,400 km^2^, mainly due to the transformation of forest and grassland ecosystems; the area of the forest ecosystem decreased by 35,000 km^2^, mainly transforming into the farmland ecosystem and grassland ecosystem; and the area of the grassland ecosystem decreased by 38,900 km^2^, mainly transforming into the forest ecosystem. The area of the water ecosystem changed little, increasing by 4000 km^2^, mainly due to the transformation of the grassland ecosystem, while the area of the settlement ecosystem increased by 63,500 km^2^, mainly from the farmland ecosystem.

Regarding the reasons, they are mainly related to the stage of China’s economic development. In different stages of economic development, the policies and measures implemented by the state are different, which leads to different characteristics of the conversion of the terrestrial ecosystem pattern in different periods. In 1999, the state officially opened the prelude to returning farmland to forest, promoting the area transformation between the farmland ecosystem and the forest and grassland ecosystems. Since 2001, there has been a period of rapid development and urbanization in China. The 10th and 11th Five-Year Plans for China’s National Economic and Social Development have put the promotion of urbanization in an important position, and a series of policies and measures have been issued, such as “Opinions on Promoting the Healthy Development of Small Towns (2000)”, “Decisions on Promoting Rural Reform and Development (2008)”, and so on, as well as the formulation of the National New Urbanization Plan (2014–2020). In addition, 2001–2015 was a period of rapid economic development in China, with a rapid increase in real estate and increasing demand for construction land, which promoted the expansion of the settlement ecosystem area during this period.

#### 3.2.2. Temporal Evolution Characteristics

TEPC and its dynamic degree in the YREB are shown in Table 4. The settlement ecosystem grew the most with a growth rate of 0.5%, an increase of 22,619 km^2^, followed by a water ecosystem, an increase of 4474 km^2^, the growth rate is 0.07%. The area of the grassland ecosystem was basically unchanged, only increasing by 856 km^2^, and the areas of the farmland ecosystem and forest ecosystem decreased by 0.02% and 0.01%, with decreased areas of 13,686 km^2^ and 13,589 km^2^, respectively. The main reasons may be as follows: In the past 20 years in China, the economy has developed rapidly, cities and towns have expanded rapidly, and the settlement ecosystem has increased rapidly. During this period, some farmland and forest resources have been occupied, and the area of the settlement ecosystem has increased but the area of the farmland and forest ecosystems has decreased. At the same time, the protection of the Yangtze River and the construction of the Belt and Road Initiative in China has led to more attention being paid to the protection of the ecological environment in the YREB, leading the areas of the water and wetland ecosystems to show a certain increasing trend.

### 3.3. Spatial Correlation Between Precipitation and the Terrestrial Ecosystem

#### 3.3.1. Bivariate Autocorrelation Characteristics of Global Space

**Figure 6:**
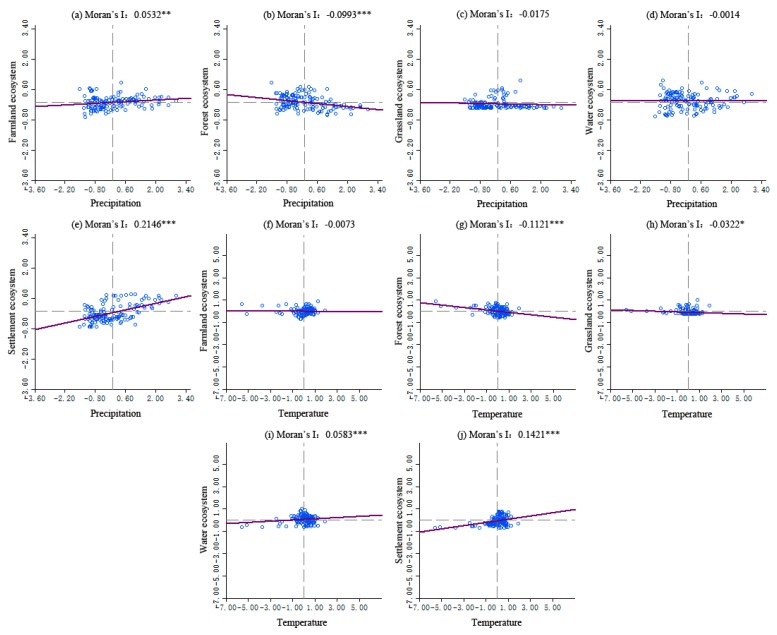
The Moran’s *I* values of precipitation and temperature with the terrestrial ecosystem in the YREB. (***, ** and * represent significant confidence levels of 99%, 95% and 90%, respectively.). (**a**) Precipitation and the farmland ecosystem; (**b**) Precipitation and the forest ecosystem; (**c**) Precipitation and the grassland ecosystem; (**d**) Precipitation and the water ecosystem; (**e**) Precipitation and the settlement ecosystem; (**f**) Temperature and the farmland ecosystem; (**g**) Temperature and the forest ecosystem; (**h**) Temperature and the grassland ecosystem; (**i**) Temperature and the water ecosystem; (**j**) Temperature and the settlement ecosystem.

The Moran’s *I* values of bivariate global spatial autocorrelation coefficients between precipitation and temperature of terrestrial ecosystems areas in the YREB are shown in Figure 6, and there are significant regional differences among different terrestrial ecosystem types. 

As for the bivariate autocorrelation between precipitation and the terrestrial ecosystem pattern, there is a significant positive correlation between precipitation and the farmland ecosystem and the settlement ecosystem, which indicates that the increase in precipitation is beneficial to the development of the farmland ecosystem to a certain extent, the agricultural output and agricultural economy are relatively better, and the development of the settlement ecosystem in precipitation-rich areas is also relatively good. There is no significant negative correlation between precipitation and the grassland ecosystem and water ecosystem. 

However, there is a significant negative correlation between precipitation and the forest ecosystem, but the negative correlation coefficient is lower. This may be related to the fact that the Yangtze River Basin is prone to geological disasters, such as collapse, landslide, debris flow, and other geological disasters induced by a large amount of precipitation, which has a certain negative impact on the development of the forest ecosystem.

As for the bivariate autocorrelation between temperature and the terrestrial ecosystem pattern, there is a significant positive correlation between temperature and the water ecosystem and settlement ecosystem, but a significant negative correlation with the forest and grassland ecosystems, and no significant negative correlation with the farmland ecosystem. The results show that the increase in temperature is beneficial to the occurrence of water ecosystem diversity to a certain extent, and the settlement ecosystem will also show diversity development because of frequent human activities, which hinders the growth of forest, grassland and other vegetation, and affects the diversity development of the ecosystem.

Compared with the relationship between temperature and precipitation and the changes in different types of terrestrial ecosystem, there is a significant negative correlation effect on the diversity of the forest ecosystem, but a significant positive correlation effect on the diversity of the settlement ecosystem. This may be due to the increase in temperature and precipitation, which to a certain extent leads to the occurrence of geological disasters in the Yangtze River Basin and the destruction of a large area of the forest ecosystem, while higher temperature and precipitation will affect the frequency of human activities, coupled with the development of urbanization and the rapid expansion of cities in China, which will promote the diversity of settlement ecosystems.

#### 3.3.2. Bivariate Autocorrelation Characteristics of Local Space

**Figure 7:**
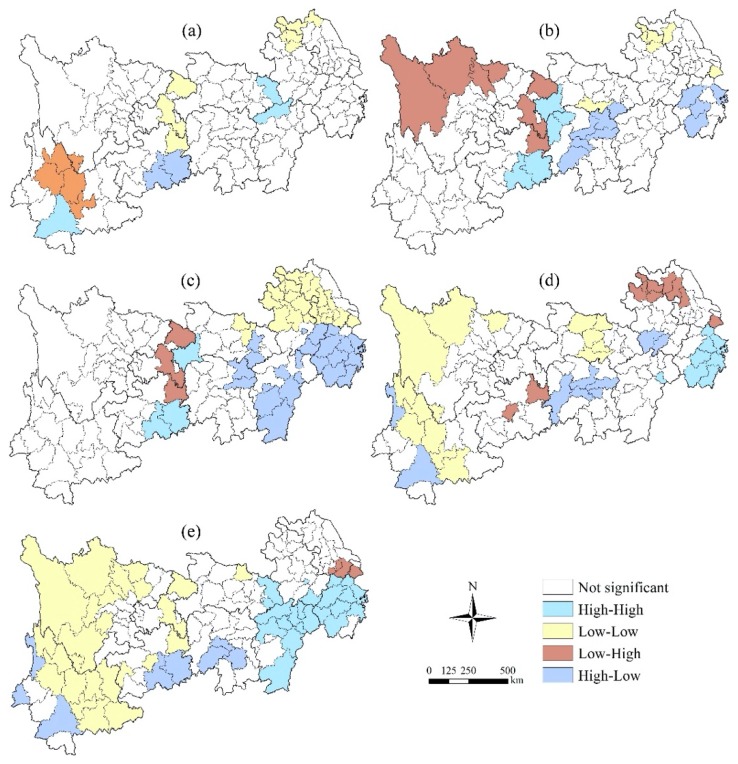
Bivariate Spatial autocorrelation characteristics between precipitation and different terrestrial ecosystems in the YREB. (**a**) Precipitation and the farmland ecosystem; (**b**) Precipitation and the forest ecosystem; (**c**) Precipitation and the grassland ecosystem; (**d**) Precipitation and the water ecosystem; (**e**) Precipitation and the settlement ecosystem.

**Figure 8:**
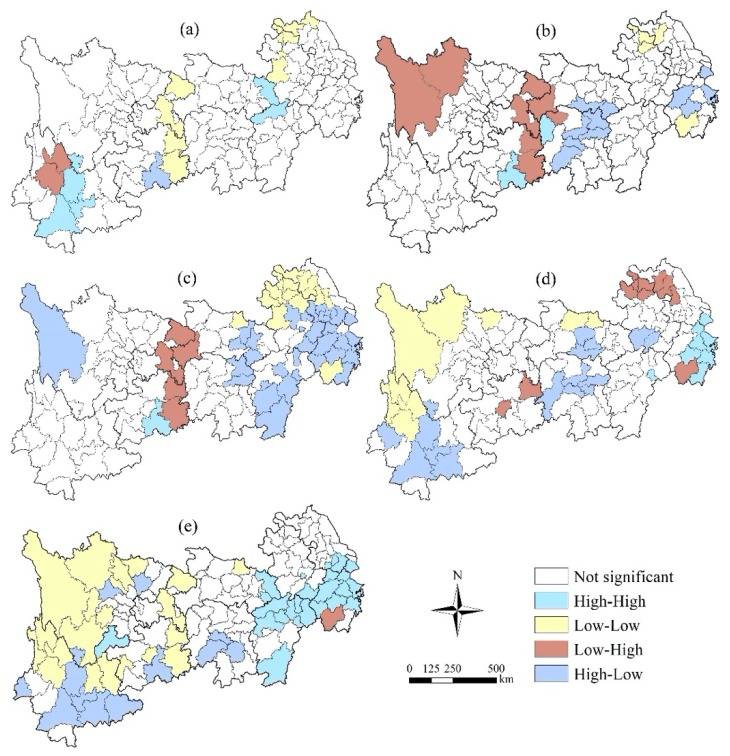
Bivariate Spatial autocorrelation characteristics between temperature and different terrestrial ecosystems in the YREB. (**a**) Temperature and the farmland ecosystem; (**b**) Temperature and the forest ecosystem; (**c**) Temperature and the grassland ecosystem; (**d**) Temperature and the water ecosystem; (**e**) Temperature and the settlement ecosystem.

On the basis of the *Z* test (*p* ≤ 0.05), the bivariate spatial autocorrelation distribution maps of precipitation, temperature and different terrestrial ecosystems are shown in Figure 7 and Figure 8. There are significant differences between precipitation and local spatial autocorrelation characteristics of different terrestrial ecosystem types. The local spatial autocorrelation can be divided into four categories: high-high, low-low, low-high and high-low, and the spatial correlation between precipitation and the terrestrial ecosystem is characterized. A high-high area indicates that the precipitation or temperature in and around the region is highly correlated with the terrestrial ecosystem. On the contrary, a low-low area indicates that the correlation between precipitation or temperature and the terrestrial ecosystem is low in the region and its surrounding areas. Low-high indicates that the correlation between precipitation or temperature and the terrestrial ecosystem in this area is lower than that in the surrounding area, while a high-low area indicates that the correlation between precipitation or temperature in this area and the terrestrial ecosystem is higher than that in the surrounding area.

Specifically, the local spatial correlation between precipitation and the farmland ecosystem shows significant scattered distribution characteristics (Figure 7), in which the low-high and high-low regions are relatively concentrated, being concentrated in the north of Yunnan province and the southern part of Guizhou province, respectively. The local spatial correlation between precipitation and the forest ecosystem is also relatively scattered, and the low-high areas are mainly distributed in the northern part of Sichuan Province. The local spatial correlation between precipitation and the grassland ecosystem is mostly in the middle and tail regions of the YREB, the low-low areas are mainly distributed in the junction zone between northern Anhui and western Jiangsu, and the high-low areas are mainly distributed in most areas of Zhejiang Province and southern Jiangxi Province. The local spatial correlation between precipitation and the water ecosystem is distributed in the head and tail regions of the YREB, in which the low-low areas are mainly distributed in the head region of western Sichuan and the central part of Yunnan Province, while the high-high areas are mainly distributed in the southeast coastal areas of Zhejiang Province. The local spatial correlation between precipitation and the settlement ecosystem is mainly reflected in the head and tail regions, in which the low-low areas are concentrated mainly in areas of Sichuan Province and eastern Yunnan Province, while the high-high areas are distributed mainly in areas of Jiangxi and Zhejiang provinces, while the distribution areas of low-high areas is smaller, only in Shanghai and southern Jiangsu.

As for the local spatial correlation between temperature and the farmland ecosystem (Figure 8), the scattered distribution characteristics are more significant, the number of regions is relatively small, there are only two cities in the high-high area, all of which are distributed in Yunnan Province, and the high-low area is only one city in Guizhou. In the local spatial correlation between temperature and the forest ecosystem, the distribution of areas with significant correlation is relatively concentrated in the central region, the low-high regions are slightly distributed in western Sichuan Province, and the low-low and high-low regions are scattered in Zhejiang Province and Jiangsu Province. The local spatial autocorrelation characteristic areas of temperature and the grassland ecosystem are mainly distributed in the tail region, in which the high-low regions are mainly distributed in most areas of Zhejiang Province and their junction with Jiangsu and Anhui, as well as the southern Jiangxi region, while the low-high areas are concentrated in Sichuan, Chongqing and eastern Guizhou, and only one city in southern Hunan exists in the high-high areas; the low-low areas are concentrated in the transition zone between western Jiangxi and northern Anhui Province. The local spatial autocorrelation areas of temperature and the water ecosystem are scattered in the upper, middle and tail regions, in which the low-low areas are mainly distributed in the head region of Sichuan Province and western Yunnan Province, and the high-high areas are mainly distributed in Shanghai city and eastern Zhejiang Province. The autocorrelation between temperature and the settlement ecosystem is widely distributed, and the low-low regions are mainly distributed in most areas of Sichuan and northern Yunnan in the head region, as well as in the east of Chongqing and Guizhou, while the high-high regions are mainly distributed in the north of Jiangxi and Zhejiang, while the low-high regions are distributed only in the southern part of Zhejiang, while the scattered distribution of high-low regions is in the middle and head regions of Yunnan.

### 3.4. Impacts of Precipitation and Temperature on Terrestrial Ecosystem Pattern Change

#### 3.4.1. Impact Utility on the Provincial Scale

Based on a total of 4655 data of temperature and precipitation for five terrestrial ecosystems in 133 prefecture-level cities of the YREB, a panel data regression analysis of precipitation and temperature was carried out to study the impact of precipitation and temperature on the terrestrial ecosystem pattern.

Taking the province as the unit, the influence coefficients of precipitation and temperature on TEPC are shown in Table 5. Generally speaking, the impact of precipitation and temperature on TEPC has significant regional characteristics, and the influence effect in the tail region is basically negative, while in the central and head regions of the YREB, both positive and negative effects exist. As far as the regional differences in human activities and economic development are concerned, the tail provinces are much more developed in their economy, the development of various industries is active, and the intensity of human activities is greater. The increase in precipitation and temperature will generally affect the behavior of human activities and the development of all kinds of industries, thus affecting the pattern change of different terrestrial ecosystems.

#### 3.4.2. Impact Utility on the City Scale

**Figure 9:**
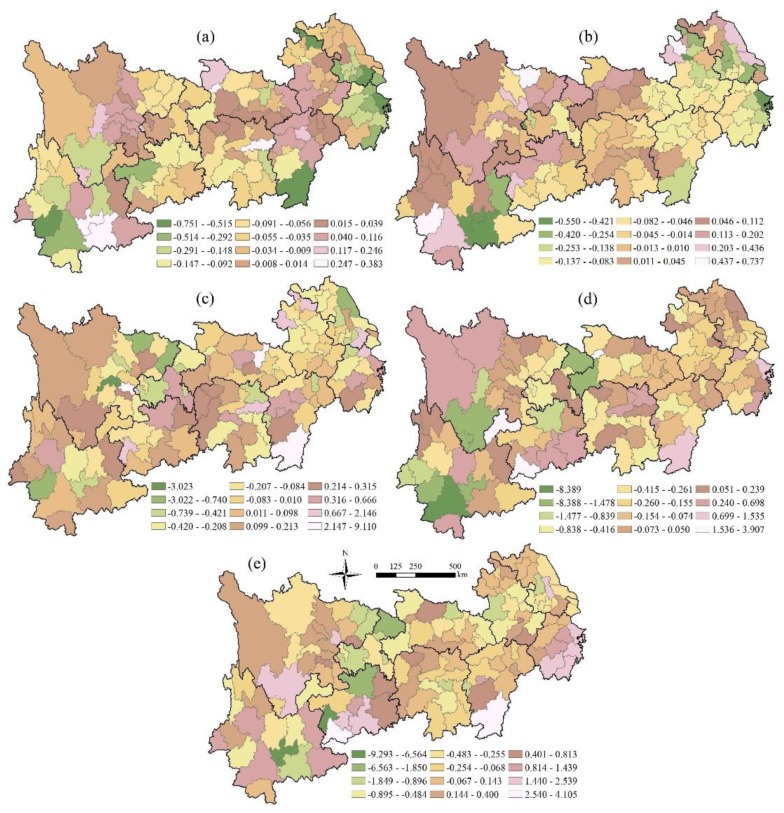
The effects of precipitation on different terrestrial ecosystems in the YREB on the city scale. (**a**) Precipitation and the farmland ecosystem; (**b**) Precipitation and the forest ecosystem; (**c**) Precipitation and the grassland ecosystem; (**d**) Precipitation and the water ecosystem; (**e**) Precipitation and the settlement ecosystem.

**Figure 10:**
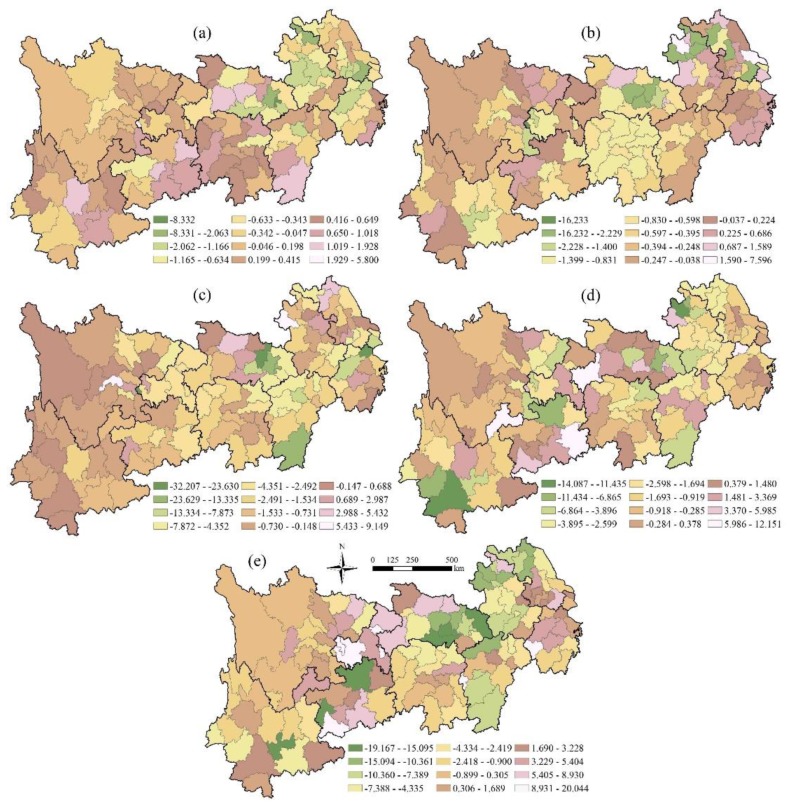
The effect of temperature on different terrestrial ecosystem patterns in the YREB on the city scale. (**a**) Temperature and the farmland ecosystem; (**b**) Temperature and the forest ecosystem; (**c**) Temperature and the grassland ecosystem; (**d**) Temperature and the water ecosystem; (**e**) Temperature and the settlement ecosystem.

In order to further clarify the impact of precipitation and temperature on TEPC, we took the prefectural city as the unit, calculated the impact of precipitation and temperature on the pattern change of different terrestrial ecosystems, and drew the spatial distribution map of impact utility as showed in Figure 9 and Figure 10. In general, precipitation and temperature have different effects on different terrestrial ecosystem patterns in different prefectural cities, showing significant spatial heterogeneity.

As for the impact of precipitation on the pattern change of the farmland ecosystem, the cities with positive influence are scattered in the south of Yunnan Province and the east of Hunan Province, and the areas with obvious negative effect are distributed in the coastal provinces of the tail region of the Yangtze River and the head region of Yunnan Province. Regarding the influence of precipitation on the changes in the forest ecosystem pattern, the most affected areas are scattered in the southeast of Yunnan Province, Anhui, and the northern part of Sichuan Province, while the negative effects areas are mainly distributed in the tail regions of Zhejiang, Anhui and Jiangxi provinces, and also scattered distribution in the southeast of Yunnan. Regarding the effect of precipitation on the changes in the grassland ecosystem pattern, the areas with the obvious negative effect are distributed in Sichuan, Yunnan, and Jiangsu, most of the negative utility areas are concentrated in the main parts of Anhui and Jiangsu and in the eastern part of Hubei Province, and the positive utility areas are mostly distributed in Hunan and Guizhou provinces. As for the effects of precipitation on the water ecosystem, the negative utility cities are mainly distributed in Chongqing city, Sichuan, and Yunnan provinces in the head region. At the same time, positive utility cities in these provinces are also widely distributed, which together affect the impact of local precipitation on the terrestrial ecosystem. The effect of precipitation on the changes in the settlement ecosystem pattern is mainly distributed in the northern part of the YREB, and the positive utility areas are mainly distributed in the south-east of the YREB and the southwest of Zhejiang Province.

The influence of temperature on the farmland ecosystem pattern is negative in Anhui, Jiangsu, and Zhejiang provinces, and the regions of positively affecting utility are mainly distributed in Yunnan, Guizhou and Hunan Provinces in the south of the YREB. As for the effects of temperature on the pattern change of the forest ecosystem, the negative effect was more significant in Hubei and Hunan provinces of the central region of the Yangtze River, and the other provinces except Hunan Province and Anhui Province had scattered distributions. Regarding the influence of temperature on the changes in the grassland ecosystem pattern, the head regions of Sichuan and Yunnan provinces have significant positive effects, while there are more negative effects in the provinces in the central and tail regions of the YREB. In terms of the effect of temperature on the water ecosystem, the number of cities with positive effect are relatively small, and the distribution is relatively scattered. The cities with negatively affecting utility are mainly distributed in the head region of the Yangtze River with Yunnan Province, Guizhou Province and Sichuan Province, and most of the negative utility areas are distributed in Anhui, Jiangsu, Zhejiang and the provinces downstream. For the effect of temperature on the settlement ecosystem pattern, the negative effect of Hubei, Anhui, and Jiangxi in the middle and tail regions of the Yangtze River is more significant and the distribution is relatively concentrated, while the positive utility areas and negative utility areas in the upstream provinces show significant cross-distribution, and the regional positive and negative effect utility characteristics are not significant.

#### 3.4.3. Analysis of Policy Impact

Due to the important strategic position of the YREB in the development of the ecological environment and social economy in China, the Chinese government has attached great importance to this region. In recent years, a series of policies and measures have been issued to promote the green, rapid and efficient development of the YREB.

With regard to national strategic planning, in September 2014, the Chinese government promulgated “The guidance on relying on the Golden Waterway to promote the Development of the YREB”, pointing out that it is necessary to rely on the Yangtze River as the golden waterway to promote the development of the YREB, build a new economic support zone for China, and optimize the overall pattern of regional development in China. The “Outline of the Development and Planning of the YREB” issued in September 2016 is a programmatic document for promoting the development of the major national strategy of the YREB, and the overall deployment of its economic development and environmental protection is made from the national level. In May 2019, the policy of “The outline of the Regional Integration Development Plan of the Yangtze River Delta” was promulgated, which is the top-level design of the national strategy for the integration development of the Yangtze River Delta at the national level, which promotes high-quality development of the social economy and ecological environment of the whole YREB. The formulation of these strategic plans has raised the protection and development of the terrestrial ecosystem of the YREB to the height of a national strategy and has provided a powerful guarantee for the sustainable and healthy development of the region.

In the aspect of ecological environment protection, “The Ecological Environment Protection Plan of the YREB” was promulgated in July 2017, and it has made a unified arrangement to improve the ecological environment of the YREB, enhance the stability of the ecosystem, restore the ecological functions of rivers, lakes and wetlands, and improve the system and mechanism of ecological environment protection. In December 2018, “The Action Plan for the Protection and Rehabilitation of the Yangtze River” was promulgated, which focuses on the effective protection of the wetland ecological functions of the main stream of the Yangtze River, the main tributaries and key lakes, so as to curb the risks to the ecological environment and improve the quality of the ecological environment. The implementation of these policies puts forward concrete guiding opinions on the protection and development of the terrestrial ecosystem in the YREB, prevents the deterioration trend of environmental pollution, protects the ecological environment effectively, and promotes the gradual improvement and effective development of the terrestrial ecosystem in the YREB.

With regard to economic support, “the implementation Plan of the Central Fiscal incentive Policy for promoting Ecological Protection and Rehabilitation of the Yangtze River Economic Belt” (2018) and “the interim measures for Investment Management within the Central Budget of the Green Development Project of the Yangtze River Economic Belt” (2018) have been promulgated and implemented, which have promoted the Yangtze River Basin to speed up the implementation of the task of ecological environment protection and control, promoted the ecological priority and green development of the YREB, and promoted the formation of the Yangtze River protection pattern as soon as possible from the aspect of financial guarantee.

At the same time, according to their own actual situation, the provinces and cities in the YREB have also formulated a series of policies and measures adapted to local realities, which effectively promote the green and healthy development of the YREB from the specific operational level. All in all, these policies and measures, such as strategic planning, ecological environment protection and economic support, have effectively curbed the development trend of the deterioration of the terrestrial ecosystem in the YREB, promoted the ecological and green development of the YREB, and promoted the coordinated development of ecology, economy and society in China.

## 4. Discussion

### 4.1. Comparison with Previous Studies

Precipitation and temperature, alongside other natural factors, are the most basic factors affecting the development of the YREB and have the status of affecting the whole ecosystem. Based on the two basic natural factors of precipitation and temperature, this paper studies their temporal and spatial variation characteristics and their influence on the area change of terrestrial ecosystems, which can provide a basic scientific reference for the protection of the ecological environment and the construction of ecological civilization in the YREB. 

Although previous studies took the factors of natural conditions such as drought [25], temperature threshold [3], and water transfer [18] into consideration, research on the direct impact of precipitation and temperature on TEPC is relatively lacking. Previous studies of the YREB were mainly focused on ecosystem services, carbon emissions, policies of ecological protection, human activities, etc. For example, Luo et al. [33] held the opinion that ecological protection policies have a significant positive effect on the ecosystem services value. The findings of Xing et al. [45] revealed that the ecological footprint in the YREB shows an ascending trend, and total-factor ecological efficiency displays a trend of “decline first and then fluctuate” at different levels, while total-factor ecological productivity shows an ascending trend. Chen et al. [32] presented current stressors, environmental and ecological status and challenges in the YREB, and offered policy recommendations on how to include ecological conservation in its development. Liu et al. [46] arrived at a result that the degree of match between urban land and the scope of human activities has an upward tendency with time in the YREB, and this may impact TEPC. 

### 4.2. Applications and Suggestions

This paper found that precipitation, temperature and terrestrial ecosystems showed significant spatial heterogeneity, and had different impacts in different regions at different times. Therefore, targeted policies and measures should be distinguished and implemented for different regions. 

For the global spatial autocorrelation between precipitation, temperature and different terrestrial ecosystems, it has a negative effect on the forest ecosystem and a positive effect on the settlement ecosystem. From 1995 to 2015, China developed rapidly in economy and urbanization, and urban areas expanded, which promoted the development of the settlement ecosystem. At the same time, the degree of human activities intensified, which easily causes temperatures to rise due to the greenhouse effect and leads to a positive correlation between temperature and the development of the settlement ecosystem. However, the increase in temperature has a certain impact on forest biodiversity, which may lead to the replacement of species, and may have a certain negative effect on the forest ecosystem. Generally speaking, precipitation is beneficial to the development of forest ecosystem biodiversity. However, excessive precipitation will lead to geological disasters such as mountain floods, landslides and so on, and the Yangtze River Basin is prone to geological disasters, so too much precipitation may have a certain inhibitory effect on the development of forest ecosystem areas.

For different regions in the YREB, the impact of precipitation and temperature on TEPC showed significant regional characteristics, and the impact in the tail region is basically negative, while in the central and head regions of the YREB, both positive and negative effects exist. There are differences in the development of different regions. The provinces in the tail region are more developed in their economy, the intensity of human activities is larger, the degree of urban expansion is higher, and the interaction between temperature, precipitation, and the development of this region is relatively obvious. In the central region, plain, hilly areas are widely distributed; the average altitude in the head region is relatively high, the regional temperature difference is larger, and climate change is more frequent. These differences lead to positive and negative effects of temperature and precipitation on this region at the same time, and lead to local regional differences.

In view of this, under the condition that the natural climate environment is difficult to change, as the YREB is an important terrestrial ecosystem area, and considering the actual situation such as topography and geomorphology, resource endowment and economic development in the head, central and tail regions of the YREB, we suggest the development of the region from the following aspects: (1) The tail region of the YREB should properly control the intensity of human activities and improve the awareness of human environmental protection, while ensuring economic development and the moderate and orderly expansion of urban areas, should reduce the negative impact of human activities on ecosystems, and should pay attention to the protection of the ecological environment. (2) The central region should reasonably use plans for all types of terrestrial ecosystems, promote the conservation and intensive use of land, and protect the ecological environment to the greatest extent while meeting the needs of human development. (3) The alpine geomorphology in the head region is diverse. From the aspects of improving environmental protection technology, improving geological disaster prevention and control technology, perfecting policy measures and so on, measures should be taken to reduce geological disasters caused by precipitation and temperature changes, to reduce the destruction of environmental quality, and to strengthen efforts to protect the originality and beauty of the terrestrial ecosystem in the YREB.

### 4.3. Limitations

This paper has several shortcomings. First, this paper studies the effects of precipitation and temperature on the area change of terrestrial ecosystems in the YREB, but there are still some factors, such as altitude, minerals, human activities, and national policies, for which it is difficult to obtain data, or difficult to carry out quantitative analysis for other objective reasons, and which this paper did not take into account. These factors directly or indirectly affect the area of terrestrial ecosystems, so more attention needs to be paid to them and more efforts made in future study. Second, in terms of the research scale, this paper studies the temporal and spatial variation characteristics of precipitation and temperature with provincial units and studies their effect on TEPC with the city as a unit. However, there is still a deficiency in terms of exploration from a more specific microscale, which would provide more accurate policy recommendations, so it is difficult to provide fine guidance for ecological protection and economic development of the YREB. Third, a spatial autocorrelation model was used to carry out the research in this paper. However, there are still some defects which need to be improved to carry out more comprehensive research. The spatial autocorrelation analysis method can only study the spatial relationship between the impact utility of temperature and precipitation on the terrestrial ecosystem, but not the differences in impact utility and the relative variation relationship of time series in different regions. 

## 5. Conclusions

This paper took the YREB as the study area from 1995 to 2015, with four 5-year sub-periods and analyzed the temporal and spatial characteristics of TEPC. STM and TEDD models were used to analyze the area transformation between farmland, forest, grassland, water and settlement terrestrial ecosystems, and the BSA model and PRM were used to study the effects of precipitation and temperature on TEPC. Several conclusions were derived.

First, during the period of 1995–2015, the basic pattern of the terrestrial ecosystem changed little and the development was relatively stable; the forest ecosystem and the farmland ecosystem accounted for 50% and 30% of the total, respectively. Area transformations between the farmland ecosystem, forest ecosystem and grassland ecosystem are much more frequent. National economic development policies and measures in different periods are the main reasons for the changes in terrestrial ecosystem areas. The temporal and spatial evolution of precipitation and temperature in different regions of the YREB, showing significant regional differences, is mainly related to the altitude, latitude and frequency of human activities.

Second, the Moran’s *I* values of bivariate global spatial autocorrelation coefficients between precipitation and temperature of terrestrial ecosystem areas in the YREB showed significant regional differences among different terrestrial ecosystem types. Both precipitation and temperature had a positive correlation with the settlement ecosystem and a negative correlation with the forest ecosystem. The local spatial correlation between precipitation or temperature and the terrestrial ecosystem showed significant scattered distribution characteristics, and also the characteristics of regional differences were significant.

Third, the impact of precipitation and temperature on the area change of terrestrial ecosystems showed significant regional characteristics, and the influence effect in the tail region was basically negative, while both positive and negative effects existed in the central and head regions on the provincial scale. As for the city scale, precipitation and temperature had different effects on different terrestrial ecosystem patterns in different prefectural cities, showing significant spatial heterogeneity.

Fourth, policies and measures for strategic planning, ecological environment protection and economic support promulgated by the Chinese government could effectively curb the development trend of the deterioration of the terrestrial ecosystem in the YREB, promote the ecological and green development of the YREB, and promote the coordinated development of ecology, the economy, and society in China.

In the future, altitude, policy, human activity, and other influencing factors need to be considered, and more data need to be obtained for a more comprehensive analysis. Based on this, a more microscopic scale, such as the county scale, needs to be studied and we hope to provide a more refined reference for the development of the YREB, and to provide more scientific reference for ecological environment protection and economic development in the YREB and even the nation. What is more, methods that can study the differences in influence utility among different regions and the relative variation relationship in time series need to be used, such as the convergence analysis method. According to this method, the spatial difference and relative development relationship between impact utilities of different regions’ temperature and precipitation on the terrestrial ecosystem pattern in the YREB can be analyzed more comprehensively, so as to provide more accurate policy suggestions for the sustainable and healthy development of the YREB.

## Figures and Tables

**Table 1:** Precipitation changes in three regions of the YREB (unit: mm).

Region of YREB	Province/City	1995	2000	2005	2010	2015	Trend Line	Average
Head region	Yunnan, Guizhou, Sichuan, Chongqing	1205	1233	1149	1133	1119	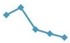	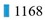
Central region	Hubei, Hunan, Jiangxi	1524	1496	1422	1695	1648	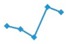	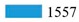
Tail region	Anhui, Shanghai, Jiangsu, Zhejiang	1177	1279	1237	1392	1542	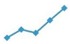	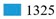
Whole regions	All eleven provinces	1302	1336	1269	1407	1437	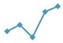	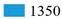

**Table 2:** Temperature changes in three regions of the YREB (°C).

Region of YREB	Province/City	1995	2000	2005	2010	2015	Trend Line	Average
Head region	Yunnan, Guizhou, Sichuan, Chongqing	13.8	13.8	14.3	14.7	14.3	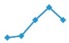	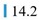
Central region	Hubei, Hunan, Jiangxi	16.9	16.9	17.2	17.4	17.5	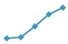	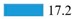
Tail region	Anhui, Shanghai, Jiangsu, Zhejiang	16.0	16.6	16.5	16.6	16.0	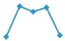	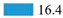
Whole regions	All eleven provinces	15.6	15.8	16.0	16.2	16.0	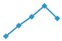	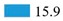

**Table 3:** Spatial transfer matrix of the terrestrial ecosystem (unit: thousand km^2^).

Later Year	Terrestrial Ecosystem	Farmland	Forest	Grassland	Water	Settlement	Total
Early Year							
1995–2000	Farmland	1266.0	205.8	151.3	34.4	88.1	479.6
	Forest	234.3	1774.6	220.5	18.7	8.3	481.8
	Grassland	167.8	221.3	2298.5	43.8	6.9	439.8
	Water	34.2	15.6	43.8	225.3	4.0	97.6
	Settlement	85.6	7.1	6.7	4.2	63.3	103.6
	Total	522.0	449.7	422.3	101.1	107.3	-
2001–2005	Farmland	1775.9	4.0	5.0	3.3	11.7	24.1
	Forest	2.3	2235.5	2.3	0.7	1.9	7.1
	Grassland	9.3	5.7	2989.2	1.4	1.0	17.4
	Water	2.6	0.3	1.3	350.0	1.1	5.2
	Settlement	0.2	0.1	0.1	0.1	172.0	0.4
	Total	14.4	10.1	8.8	5.5	15.6	-
2006–2010	Farmland	1781.9	1.8	1.3	1.1	7.1	11.3
	Forest	1.0	2242.1	1.3	0.3	1.0	3.6
	Grassland	2.5	2.1	2994.1	0.7	0.5	5.8
	Water	0.9	0.1	0.6	354.8	0.5	2.1
	Settlement	0.1	0.0	0.0	0.1	188.1	0.3
	Total	4.6	4.0	3.2	2.1	9.2	-
2011–2015	Farmland	1768.5	1.1	1.5	1.6	14.9	19.0
	Forest	2.4	2237.3	2.1	0.6	3.6	8.8
	Grassland	7.8	1.0	2983.5	2.0	3.9	14.7
	Water	2.0	0.1	0.9	352.9	1.1	4.2
	Settlement	1.2	0.3	0.2	0.2	196.0	1.8
	Total	13.5	2.4	4.6	4.4	23.5	-
1995–2015	Farmland	1237.6	206.9	149.8	36.5	115.3	508.5
	Forest	232.8	1767.4	221.5	19.8	14.8	488.9
	Grassland	178.4	224.9	2277.4	45.4	12.1	460.8
	Water	35.9	15.4	44.0	220.8	6.5	101.8
	Settlement	81.8	6.8	6.6	4.2	67.7	85.2
	Total	528.9	453.9	421.9	105.8	148.7	-

**Table 4:** Terrestrial ecosystem pattern change and its dynamic degree in the YREB.

Ecosystem	1995–2000		2000–2005		2005–2010		2010–2015		1995–2015	
	AREA (km^2^)	TEDD (%)	AREA (km^2^)	TEDD (%)	AREA (km^2^)	TEDD (%)	AREA (km^2^)	TEDD (%)	AREA (km^2^)	TEDD (%)
Farmland ecosystem	5769	0.91	−6522	−1.02	−4953	−0.78	−7980	−1.27	−13,686	−0.02
Forest ecosystem	−12,351	−1.30	1128	0.12	414	0.04	−2780	−0.30	−13,589	−0.01
Grassland ecosystem	3088	0.91	−1414	−0.41	−504	−0.15	−314	−0.09	856	0.00
Water ecosystem	1784	2.93	1257	2.01	478	0.75	955	1.48	4474	0.07
Settlement ecosystem	2211	4.93	5645	12.00	4520	8.58	10,243	17.90	22,619	0.50

**Table 5:** The impacts of precipitation and temperature on TEPC in the YREB on the provincial scale.

Region of YREB	Province/City	*x1*	*x2*	*x3*	*x4*	*x5*	*y1*	*y2*	*y3*	*y4*	*y5*
Head region	Sichuan	0.017	0.127	0.110	−0.224	0.004	−0.364	−0.280	−0.958	0.140	0.334
	Yunnan	−0.052	0.408	0.028	−0.386	0.007	−0.507	−0.571	−1.660	−0.252	−1.757
	Chongqing	0.036	1.299	−0.137	−3.804	0.221	−7.755	−0.457	0.375	−0.816	8.400
	Guizhou	−0.114	0.347	0.045	−0.281	0.239	−1.363	0.300	0.965	−0.202	−0.756
Central region	Hubei	0.015	−0.792	−0.005	−0.746	0.632	−5.128	−0.070	1.131	−0.211	−5.306
	Hunan	−0.016	0.391	−0.004	−0.829	0.125	−1.918	−0.124	−0.954	−0.146	−1.649
	Jiangxi	−0.024	0.006	−0.058	−0.410	0.640	−4.689	0.022	−0.996	0.321	−3.069
Tail region	Anhui	−0.041	−0.772	−0.042	−0.015	0.114	−0.954	−0.152	−2.786	−0.114	−5.613
	Jiangsu	−0.134	−0.629	−0.052	−0.111	−0.051	−0.515	−0.051	−1.587	−0.076	−2.456
	Shanghai	−0.252	−0.530	−0.114	0.074	−0.030	−6.407	−0.231	−0.167	−0.388	1.258
	Zhejiang	−0.264	−0.431	−0.138	−0.010	0.180	−5.300	0.152	0.259	1.056	2.107

Note: *x1, x2, x3, x4* and *x5* represent the impacts of precipitation on pattern changes of the farmland ecosystem, forest ecosystem, grassland ecosystem, water ecosystem and settlement ecosystem, respectively. *y1, y2, y3, y4* and *y5* represent the impacts of temperature on pattern changes of the farmland ecosystem, forest ecosystem, grassland ecosystem, water ecosystem and settlement ecosystem, respectively.
